# Two Cases of Levetiracetam-Induced Rhabdomyolysis With Low Levetiracetam Blood Concentrations

**DOI:** 10.7759/cureus.80877

**Published:** 2025-03-20

**Authors:** Kentaro Kodama, Toru Imai, Tsukasa Kuwana, Susumu Ootsuka, Kosaku Kinoshita

**Affiliations:** 1 Department of Pharmacy, Nihon University Itabashi Hospital, Tokyo, JPN; 2 Department of Acute Medicine, Nihon University School of Medicine, Tokyo, JPN

**Keywords:** antiepileptic drug, creatine kinase, lacosamide, levetiracetam, rhabdomyolysis, synaptic vesicle glycoprotein 2a

## Abstract

Levetiracetam is an antiepileptic drug used to treat tonic-clonic convulsions and partial seizures. In general, levetiracetam is well tolerated; however, rhabdomyolysis has been reported as a rare side effect. The underlying mechanism is unclear. This case report describes two cases of rhabdomyolysis during levetiracetam treatment for convulsive status epilepticus, both of whom developed rhabdomyolysis a few days after starting levetiracetam. When levetiracetam was replaced with lacosamide, creatinine kinase decreased to the normal range within approximately 10 days. Furthermore, blood levels of levetiracetam were measured at the onset of rhabdomyolysis, and the results suggest that its adverse effects may not be correlated with its levels in the blood. Monitoring creatinine kinase is important for early detection of levetiracetam-induced rhabdomyolysis.

## Introduction

Levetiracetam (LEV) binds to synaptic vesicle glycoprotein 2A (SV2A) and modulates its function [1]. It is an antiepileptic drug used for partial seizure, myoclonic seizure, and tonic-clonic seizure treatment [2,3]. LEV, a second-generation antiepileptic drug, is not more effective than first-generation antiepileptic drugs [4] but has fewer drug interactions than first-generation antiepileptic drugs such as phenobarbital, phenytoin, and carbamazepine [5]. The side effects of LEV include somnolence, dizziness, and irritability [2,3]. However, LEV is widely used in clinical practice because it is safe and has few serious side effects. LEV causes rhabdomyolysis, a serious but infrequent side effect [6]; however, the underlying mechanism remains unclear. Thus, it is imperative to elucidate the underlying mechanism to ensure the safe continuation of drug therapy. Some drugs that cause rhabdomyolysis are known to be toxic in a dose-dependent manner. For example, daptomycin and haloperidol have been shown to increase creatinine kinase (CK) levels at high blood concentrations [7,8]. The therapeutic range of LEV is 12-46 µg/mL [9]. However, to our knowledge, there are no reports on blood concentrations measured in cases of LEV-induced rhabdomyolysis, and the relationship between LEV blood concentrations and rhabdomyolysis is unclear. Herein, we describe two cases of LEV-associated rhabdomyolysis and LEV blood levels measured at the onset of rhabdomyolysis.

## Case presentation

Case 1

**Figure 1:**
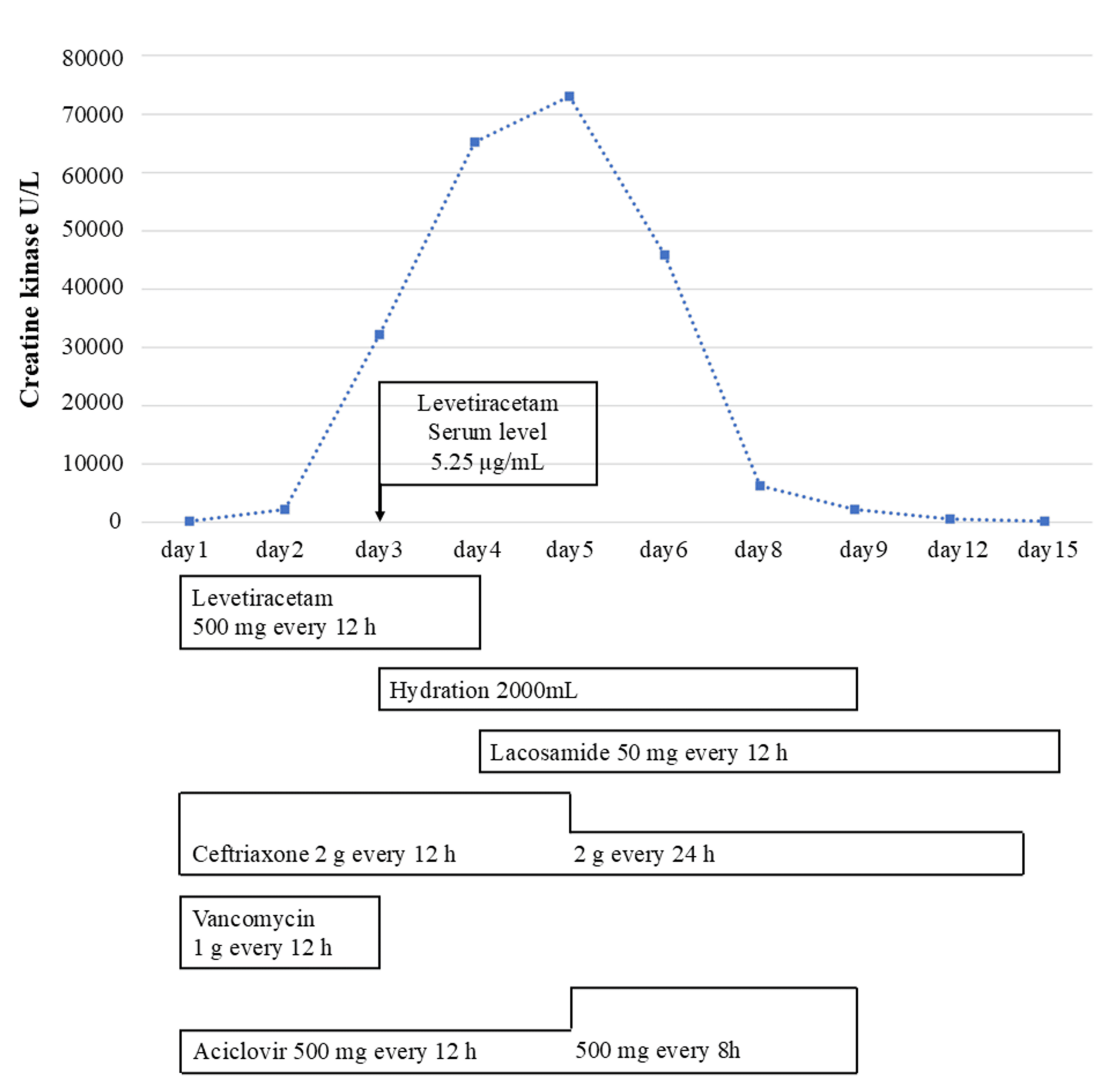
Creatine kinase and levetiracetam serum levels in case 1

A male patient in his 30s was admitted to the emergency department (ED) with loss of consciousness. The only medical history was febrile convulsions in childhood, but no medications were regularly administered. On admission, his vital signs were as follows: blood pressure 132/76 mmHg, heart rate 168 beats/minute (sinus tachycardia), respiratory rate 30 breaths/minute, saturation of percutaneous oxygen (SpO_2_) 87% (with a reservoir mask at 10 L/minute), Glasgow Coma Scale (GCS) 9 (E4V1M4), axillary temperature 39.2°C, pupil diameter 3.0/3.0 mm, bilateral contralateral light reflexes were rapid, and co-polarization was observed. The patient was intubated to maintain the airway for a prolonged disturbance of consciousness. Arterial blood gas analysis showed the following: pH 7.028, arterial partial pressure of carbon dioxide 35.9 mmHg, arterial partial pressure of oxygen 69.3 mmHg, bicarbonate 9.0 mmol/L, base excess (BE) -19.6 mmol/L, and lactate 17.0 mmol/L (Table 1). The patient had lactic acidosis, and LEV 500 mg was administered, considering convulsive seizures as one of the causes of impaired consciousness. Simple computed tomography (CT) of the head revealed no cerebral hemorrhage. Chest CT showed pneumonia in the lower left lobe. A urinary drug screening test was negative for all drugs, including amphetamines and cocaine. Blood tests showed moderate renal dysfunction with an estimated creatinine clearance (Ccr) of 48.8 mL/minute, hyperphosphatemia (7.4 mg/dL), and hypermagnesemia (3.6 mg/dL) (Table 2). After admission to the intensive care unit (ICU), a lumbar puncture was performed to investigate the cause of consciousness disturbance, considering the possibility of a central nervous system infection. Cerebrospinal fluid (CSF) was clear and colorless with an initial pressure of 28 mmH_2_O, cell count of 6/μL, CSF sugar of 118 mg/dL (blood sugar 231 mg/dL), and CSF protein of 413 mg/dL (Table 3). Bacterial meningitis was negative, but ceftriaxone (CTRX) and vancomycin (VCM) were initiated as empirical therapies until the culture results were clear. In addition to this, treatment with acyclovir was initiated to manage possible viral meningitis. A generalized clonic seizure lasting four minutes was observed two hours after admission to the ICU, which resolved after administering 10 mg of diazepam. Thereafter, LEV 500 mg, which was initiated in the ED, was administered every 12 hours. Electroencephalography (EEG) on the second day revealed a high frequency of high-amplitude slow waves with frontal dominance; however, no seizures were observed. His consciousness level was GCS E3VTM5, renal function improved with a 73.1 mL/minute Ccr, and phosphorus and magnesium levels were within the normal range. On day 3, the patient’s general condition improved; however, his CK level increased from 207 U/L (day 1) to 32,124 U/L (Figure 1). On the same day, the patient was hydrated with 2,000 mL/day of acetated Ringer’s solution and had sufficient urinary output. The blood trough concentration of LEV was 5.25 µg/mL (therapeutic range: 12-46 μg/mL). On day 4, the patient was weaned off the ventilator because his level of consciousness improved to GCS E4VTM6. Magnetic resonance imaging (MRI) of the head revealed no encephalitis or encephalopathy. The CK level on day 4 further increased to 65,207 U/L, but there was no muscle pain. Therefore, we suspected drug-induced rhabdomyolysis and considered stopping or changing the drug. CSF culture was negative, and sputum culture revealed penicillin-sensitive *Streptococcus pneumoniae*. Therefore, VCM treatment was deemed unnecessary and discontinued after treatment day 3. LEV was discontinued and changed to 50 mg lacosamide (LCM) every 12 hours. On day 5, the CTRX dose was reduced from 4 g/day for meningitis to 2 g/day for pneumonia. After two days of VCM discontinuation and one day of LEV discontinuation, the CK value peaked at 73,007 U/L on day 5 and 45,813 U/L on day 6. It decreased to 6,295 U/L on day 8 and improved to the normal range on day 12. Blood culture on the first day was negative, and herpes simplex and varicella-zoster virus deoxyribonucleic acid in the spinal fluid were negative on day 5. The cause of disturbance in consciousness was thought to be convulsive status epilepticus. After a generalized clonic seizure immediately after admission to the ICU, the seizures did not recur, and the patient was discharged on day 20.

**Table 1:** Arterial blood gas at the time of admission in case 1 PaCO_2_ - arterial partial pressure of carbon dioxide; PaO_2_ - arterial partial pressure of oxygen

Analyte	Value	Reference range
pH	7.028	7.350-7.450
PaCO_2_(mmHg)	35.9	35-45
PaO_2 _(mmHg)	69.3	80-100
Bicarbonate (mmol/L)	9.0	22-26
Base excess (mmol/L)	-19.6	-2.0-2.0
Lactate (mmol/L)	17.0	0.5-2.0
Glucose (mg/dL)	214	70-100

**Table 2:** Basic metabolic panel at the time of admission in case 1 BUN - blood urea nitrogen

Laboratory test	Result value	Reference range
Creatine kinase (U/L)	207	59-248
BUN (mg/dL)	27.9	8.0-20
Creatine (mg/dL)	1.54	0.65-1.07
Sodium (mmol/L)	141	138-145
Potassium (mmol/L)	4.4	3.6-4.8
Chloride (mmol/L)	97	101-108
Calcium (mg/dL)	9.4	8.8-10.1
Phosphorus (mg/dL)	7.4	2.7-4.6
Magnesium (mg/dL)	3.6	1.7-2.3

**Table 3:** Cerebrospinal fluid findings at the time of admission in case 1

Cerebrospinal fluid analysis findings	Result value	Reference range
Opening pressure (mmH_2_O)	28	10-20
Cell count (per μL)	6	<5
Sugar (mg/dL)	118	50-75
Protein (mg/dL)	413	10-40

Case 2

**Figure 2:**
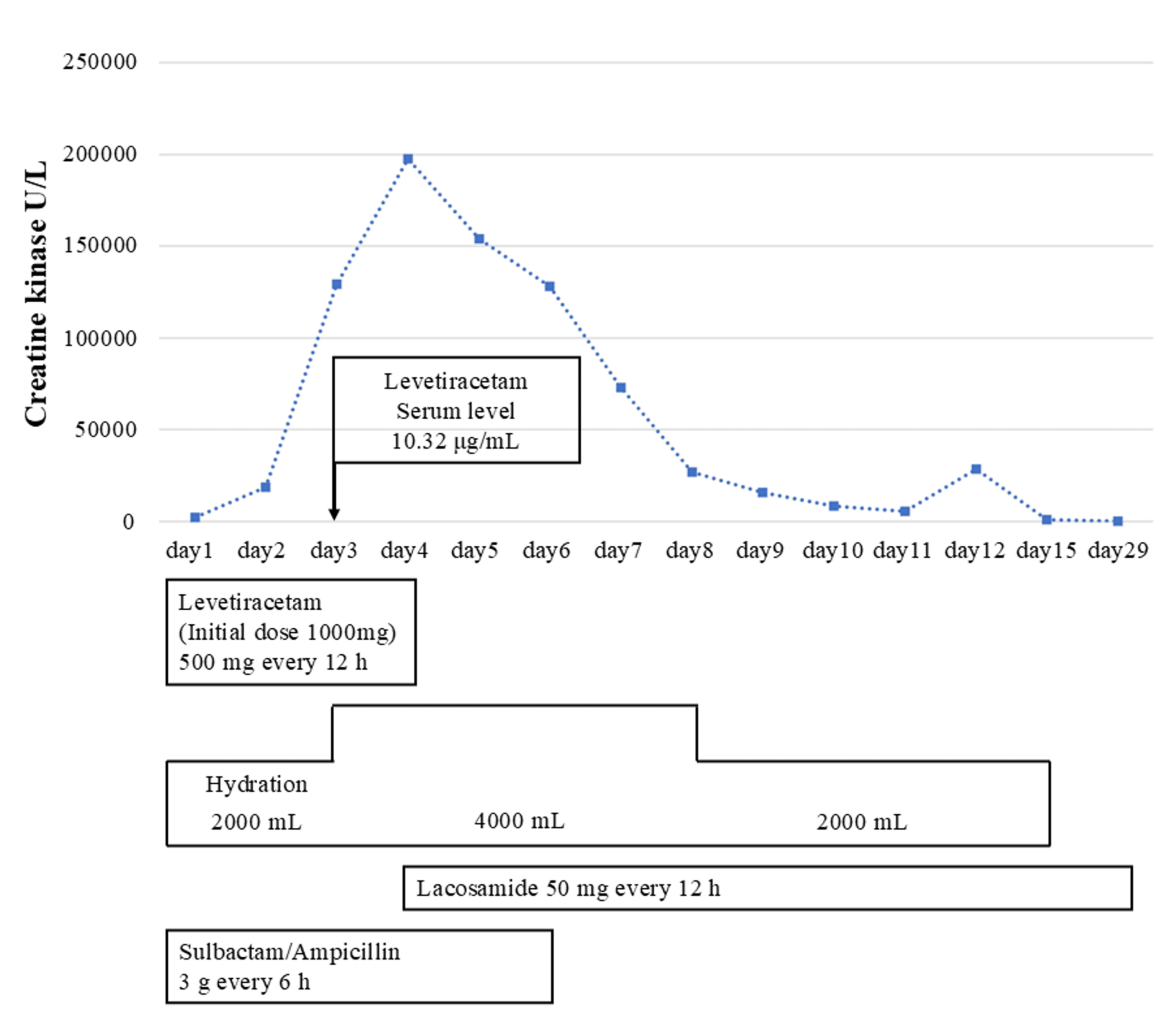
Creatine kinase and levetiracetam serum levels in case 2

A male patient in his 20s was admitted to the ED due to prolonged loss of consciousness after a one-minute tonic seizure. His history included pervasive developmental disorders and flunitrazepam, lorazepam, triazolam, and valproic acid administration. On admission, his vital signs were as follows: blood pressure 120/88 mmHg, heart rate 132 beats/minute (sinus tachycardia), respiratory rate 36 breaths/minute, SpO_2_ 88% (with a reservoir mask at 10 L/minute), GCS 6 (E1V1M4), axillary temperature 36.5°C, pupil diameter 3.0/3.0 mm, slow bilateral light reflex, and bilateral elbows with subcutaneous hemorrhages. Two minutes after arrival at the ED, a one-minute generalized tonic convulsion occurred. Diazepam (10 mg) was administered for its anti-epileptic effect. Subsequently, LEV was initiated. Venous blood gas analysis showed the following: pH 6.886, mixed venous carbon dioxide 86.8 mmHg, mixed venous oxygen pressure 38.6 mmHg, bicarbonate 15.6 mmol/L, BE -15.7 mmol/L, lactate 16.0 mmol/L, and blood glucose 68 mg/dL, indicating mixed acidosis and hypoglycemia (Table 4). The patient was administered 40 mL of 20% glucose for hypoglycemia; however, his consciousness did not improve; he was intubated to secure his airway. Blood tests showed Ccr 71.7mL/minute, mild renal dysfunction, hypernatremia (152 mEq/L), hyperkalemia (5.5 mEq/L), hyperphosphatemia (11.4 mg/L), hypermagnesemia (4.3 mg/dL), and elevated CK at 2,340 U/L (Table 5). A chest CT revealed pneumonia, and aspiration pneumonia was diagnosed. A head CT showed a mandibular fracture and subdural hematoma. Post-admission to the ICU, hydration with 2,000 mL/day of acetated Ringer’s solution, 500 mg LEV, and 3 g ampicillin/sulbactam (APBC/SBT) for aspiration pneumonia were administered every 12 and six hours, respectively. Continuous midazolam was administered for sedation during ventilation. A head CT on day 2 of the patient’s illness did not reveal any enlargement of the subdural hematoma. The qualitative urine drug screening test was positive for benzodiazepines and negative for amphetamines and cocaine. The CK level increased to 18,532 U/L, and hydration continued (Figure 2). Electrolyte abnormalities improved, but renal function remained unchanged. On day 3, the patient’s consciousness level improved to GCS E4VTM5; thus, midazolam was discontinued, and he was weaned off the ventilator. Hydration was intensified to 4,000 mL/day because the CK level further increased to 129,496 U/L despite adequate urine output with continued hydration. The patient did not complain of myalgia. The blood trough concentration of LEV was 10.32 µg/mL. On day 4, the CK level further increased to 197,498 U/L. When drug-induced rhabdomyolysis was suspected, we considered discontinuing or changing the drug. LEV was discontinued and changed to 50 mg LCM every 12 hours. The other drugs were continued as therapeutically necessary. EEG showed no epileptic waves, and a head MRI showed no abnormal findings on the fifth day. CK levels were 153,863 U/L on day 5, after one day of LEV discontinuation, 128,205 U/L on day 6, and 26,806 U/L on day 8. CK improved to the normal range on day 15. APBC/SBT for aspiration pneumonia was completed on day 6. The cause of disturbance in consciousness was thought to be convulsive status epilepticus. Generalized tonic seizures did not recur in the ED, and the patient was discharged on day 16.

**Table 4:** Venous blood gas at the time of admission in case 2 PvCO_2_ - mixed venous carbon dioxide; PvO_2_ - mixed venous oxygen pressure

Analyte	Value	Reference range
pH	6.886	7.310-7.410
PvCO_2_(mmHg)	86.8	41-51
PvO_2 _(mmHg)	38.6	30-40
Bicarbonate (mmol/L)	15.6	22-26
Base excess (mmol/L)	-15.7	-2.0-2.0
Lactate (mmol/L)	16.0	0.5-2.0
Glucose (mg/dL)	68	70-100

**Table 5:** Basic metabolic panel at the time of admission in case 2 BUN - blood urea nitrogen

Laboratory test	Result value	Reference range
Creatine kinase (U/L)	2,340	59-248
BUN (mg/dL)	16.5	8.0-20
Creatine (mg/dL)	2.11	0.65-1.07
Sodium (mmol/L)	152	138-145
Potassium (mmol/L)	5.5	3.6-4.8
Chloride (mmol/L)	108	101-108
Calcium (mg/dL)	9.5	8.8-10.1
Phosphorus (mg/dL)	11.4	2.7-4.6
Magnesium (mg/dL)	4.3	1.7-2.3

## Discussion

Two cases of rhabdomyolysis with a sudden increase in CK levels at the beginning of LEV administration improved after discontinuation. In both patients, blood LEV concentrations were low at the onset of rhabdomyolysis. This is the first case report of rhabdomyolysis in which blood LEV concentrations were measured at disease onset. Rhabdomyolysis is an increase in serum CK levels of >10 times the normal upper limit [10]. Major causes are illegal drugs, alcohol, medications, muscle diseases, trauma, seizures, infections, and electrolyte abnormalities (hypernatremia, hyponatremia, hypokalemia, hypophosphatemia, and hypocalcemia) [11,12]. In these two cases, no illegal drugs causing rhabdomyolysis were detected in the urinary drug screening test, and the osmotic pressure gap was <10 mOsm/kg H_2_O. Additionally, there was no history of metabolic disorders, lipid metabolic disorders, myopathy, mitochondrial disorders, muscular dystrophy, or other diseases that could have caused rhabdomyolysis.

In case 1, a sudden increase in CK level was observed from day 3 of hospitalization, fulfilling the definition of rhabdomyolysis [10]. Despite the initiation of appropriate infusion therapy, the peak CK level on day 4 elevated to approximately 352 times the value on admission. We examined the influence of convulsive seizures on rhabdomyolysis. The rate of increase in CK levels due to convulsive seizures was greater in tonic-clonic seizures than in partial seizures. CK values increase an average of 7.5-fold for one to four seizures and an average of 38-fold for ≥5 seizures [13]. The mean time to peak CK level was 1.7 days. However, the patient’s consciousness level improved on the second day, and convulsions did not recur. Therefore, we considered the possibility of rhabdomyolysis due to convulsive seizures to be low. Next, VCM and LEV were discontinued, considering drug-related influences. There have been reports of LEV-induced rhabdomyolysis [14]. However, there have been no reports of VCM-induced rhabdomyolysis, and we considered LEV as a suspected drug. In drug-induced rhabdomyolysis, symptoms improve after discontinuation of the causative drug [15]. The time to peak CK in LEV-induced rhabdomyolysis was previously reported to be four to five days in most cases [14]. Furthermore, the time to peak CK after discontinuation of LEV was previously reported to be one day [6]. The CK level trends in this case were consistent with these previous reports, and the CK levels improved with LEV discontinuation, which resulted in a positive dechallenge. Therefore, rhabdomyolysis was possibly caused by LEV. Infection is also a possible cause of rhabdomyolysis. In this case, sputum culture was positive for penicillin-susceptible *S. pneumoniae*. There are several reports of rhabdomyolysis caused by pneumococcal infection; however, in these reports, the CK value peaked on day 1, and the blood culture was positive for *S. pneumoniae* [16,17]. In our case, the CK values and negative blood culture trend suggested that the rhabdomyolysis was unlikely to have originated from infectious agents. Thus, in case 1, after careful exclusion of other conditions that could have caused rhabdomyolysis, we concluded that LEV-induced rhabdomyolysis was the most likely cause.

In case 2, elevated CK levels were observed at the time of admission. Despite appropriate infusion therapy from day 1, the CK levels increased on day 2, fulfilling the diagnostic criteria for rhabdomyolysis. Furthermore, the peak CK level on day 4 was approximately 84 times higher than that on admission. We considered the possibility that rhabdomyolysis was associated with convulsive seizures. However, the seizures did not recur after the administration of antiepileptic drugs. Furthermore, the patient’s consciousness level improved over time. Therefore, we considered the possibility of rhabdomyolysis due to convulsive status epilepticus to be low, as in case 1. Next, we considered the drug-related involvement and discontinued LEV. As in case 1, the CK values in case 2 were similar to previous reports of LEV-induced rhabdomyolysis [6,14]; the CK values worsened with the continuation of LEV and improved with its discontinuation, resulting in a positive dechallenge. These findings suggested a high possibility of LEV-induced rhabdomyolysis. Severe hypernatremia (184 mEq/L) also causes rhabdomyolysis [18]. However, in this case, there was no severe hypernatremia, and the possibility of rhabdomyolysis due to electrolyte abnormalities was low. Furthermore, mandibular fractures and subcutaneous hemorrhages were observed around both elbows. Rhabdomyolysis occurs in patients with numerous contusions and subcutaneous hemorrhages in the upper body [19]. However, we did not find any reports of rhabdomyolysis in patients with minor subcutaneous hemorrhages. In this case, the extent of subcutaneous bleeding was limited, and the possibility of rhabdomyolysis due to trauma was considered low. Thus, in case 2, after excluding other conditions that could have caused rhabdomyolysis, we concluded that LEV was the most likely cause of rhabdomyolysis.

The half-life of LEV is 7 ± 1 hours [20]. It takes two days for blood levels to reach a steady state. In our cases, blood levels were measured on day 3 after LEV initiation. The blood concentrations in these cases were below the general LEV therapeutic range of 12-46 µg/mL [9]. In other words, in the cases we have experienced, blood levels of LEV were low at the onset of rhabdomyolysis. Therefore, we suggest that its toxicological mechanism may be dose-independent and develop early in the initiation of treatment. LEV-induced rhabdomyolysis developed early in the initiation of administration, similar to this report [6]. Drug-induced rhabdomyolysis can be dose-dependent or dose-independent based on its pathogenic mechanism. Statins cause rhabdomyolysis in a dose-dependent manner [21]. The toxic mechanism may involve decreased coenzyme Q10 content in human skeletal muscle and oxidative phosphorylation in mitochondria [22]. However, no direct cytotoxicity to skeletal muscle has been reported for LEV, but an improving effect on mitochondrial function was suggested [23]. Based on these findings, the dose-independent mechanism by which LEV causes rhabdomyolysis may differ from that by statins. LEV increases blood levels in patients with renal insufficiency, but it is safe to use in these patients [24]. This report supports the hypothesis that the mechanism of LEV-induced rhabdomyolysis is dose-independent. Inhaled anesthetics cause rhabdomyolysis via malignant hyperthermia [25]. The mechanism may involve enhanced calcium release due to ryanodine receptor 1 gene mutations in the skeletal muscle [26]. Therefore, it may occur in a dose-independent manner, depending on factors such as patient susceptibility and genetic background. Additionally, donepezil, a cholinesterase inhibitor, causes rhabdomyolysis right at the onset of administration, possibly in a dose-independent manner [27], whereby donepezil increases acetylcholine levels and induces abnormal muscle contractions, leading to rhabdomyolysis. LEV also enhances cholinergic function [28]. Therefore, it may have a similar rhabdomyolysis-inducing mechanism to that of donepezil. Furthermore, SV2A is expressed in the motor nerve terminals of slow muscle fibers in mice [29]. LEV may cause rhabdomyolysis through an unidentified mechanism mediated by SV2A. Factors such as patient background, genetic mutations, and new mechanisms may be involved in the development of LEV-induced rhabdomyolysis. Elucidating these factors is important to further improve the safety of LEV.

## Conclusions

Rhabdomyolysis can lead to severe complications, such as acute renal failure, hyperkalemia, and metabolic acidosis. CK levels should be regularly measured when LEV is administered to detect rhabdomyolysis at an early stage and provide appropriate treatment. The mechanism of LEV-induced rhabdomyolysis should be confirmed in more detail through further case accumulation and research, leading to the implementation of safe drug treatments.
